# Supplementary material for: A Likelihood Approach for Real-Time Calibration of Stochastic Compartmental Epidemic Models
Source: PLoS Comput Biol. 2017 Jan 17;13(1):e1005257. doi: 10.1371/journal.pcbi.1005257 (PMC5240920; doi:10.1371/journal.pcbi.1005257)
Supplement: S1 File — (TAR.GZ) [file pcbi.1005257.s014.tar.gz › HSPH_Online-SI-Revision/PF08/n10-severe/n10-severe_PF2_table4.pdf]

1	newly 3 weeks spec	197.	284.79	{248., 317.}	44.6041	0.158058	newly 3 weeks cumul	743.	972.64	{875., 1055.}	31.0067	0.116312	newly 5 weeks spec	99.	208.97	{176., 243.}	111.081	0.320361	newly 5 weeks cumul	972.	1424.22	{1283., 1570.}	46.5247	0.16444	newly 47 weeks cumul	1325.	2483.71	{2054., 2859.}	87.4498	0.269743
2	newly 3 weeks spec	183.	352.34	{306., 409.}	92.5355	0.282265	newly 3 weeks cumul	725.	1223.25	{1111., 1381.}	68.7241	0.225921	newly 5 weeks spec	116.	245.7	{209., 295.}	111.81	0.322505	newly 5 weeks cumul	1003.	1760.39	{1585., 1983.}	75.5125	0.242874	newly 49 weeks cumul	1262.	2855.15	{2513., 3301.}	126.24	0.352315
3	newly 3 weeks spec	87.	319.39	{244., 406.}	267.115	0.556287	newly 3 weeks cumul	412.	1124.62	{901., 1389.}	172.966	0.43009	newly 5 weeks spec	40.	217.79	{155., 288.}	444.475	0.725115	newly 5 weeks cumul	486.	1607.74	{1262., 2005.}	230.811	0.512684	newly 51 weeks cumul	514.	2494.73	{1853., 3281.}	385.356	0.675268
4	newly 3 weeks spec	158.	274.2	{249., 296.}	73.5443	0.238427	newly 3 weeks cumul	634.	931.99	{890., 972.}	47.0016	0.167015	newly 5 weeks spec	66.	204.61	{183., 229.}	210.015	0.489668	newly 5 weeks cumul	805.	1377.78	{1316., 1433.}	71.1528	0.233077	newly 46 weeks cumul	946.	2504.28	{2330., 2645.}	164.723	0.422258
5	newly 3 weeks spec	156.	284.7	{261., 319.}	82.5	0.260017	newly 3 weeks cumul	639.	967.81	{915., 1023.}	51.457	0.179854	newly 5 weeks spec	112.	214.85	{190., 242.}	91.8304	0.280801	newly 5 weeks cumul	896.	1430.45	{1342., 1516.}	59.6484	0.202688	newly 46 weeks cumul	1167.	2578.93	{2307., 2763.}	120.988	0.343261
6	newly 3 weeks spec	63.	300.36	{264., 332.}	376.762	0.676842	newly 3 weeks cumul	405.	997.82	{910., 1052.}	146.375	0.391036	newly 5 weeks spec	51.	229.19	{198., 260.}	349.392	0.650488	newly 5 weeks cumul	521.	1491.84	{1369., 1575.}	186.342	0.456249	newly 47 weeks cumul	643.	2793.93	{2444., 3012.}	334.515	0.636771
7	newly 3 weeks spec	115.	229.01	{170., 270.}	99.1391	0.293004	newly 3 weeks cumul	383.	774.96	{598., 877.}	102.339	0.301252	newly 5 weeks spec	76.	177.8	{124., 209.}	133.947	0.360605	newly 5 weeks cumul	548.	1155.5	{880., 1332.}	110.858	0.318698	newly 46 weeks cumul	668.	2198.32	{1470., 2632.}	229.09	0.508022
8	newly 3 weeks spec	100.	336.01	{286., 370.}	236.01	0.524422	newly 3 weeks cumul	467.	1178.2	{1052., 1266.}	152.291	0.400706	newly 5 weeks spec	34.	228.81	{198., 260.}	572.971	0.825416	newly 5 weeks cumul	561.	1684.97	{1500., 1817.}	200.351	0.476295	newly 50 weeks cumul	610.	2677.23	{2368., 2963.}	338.89	0.640223
9	newly 3 weeks spec	135.	392.81	{308., 473.}	190.97	0.458425	newly 3 weeks cumul	764.	1442.64	{1200., 1696.}	88.8272	0.272678	newly 5 weeks spec	57.	245.1	{172., 317.}	330.	0.621702	newly 5 weeks cumul	905.	2002.01	{1603., 2369.}	121.217	0.340306	newly 54 weeks cumul	1026.	2858.01	{2122., 3569.}	178.558	0.436533
10	newly 3 weeks spec	69.	308.86	{231., 370.}	347.623	0.643871	newly 3 weeks cumul	417.	1169.64	{963., 1381.}	180.489	0.444089	newly 5 weeks spec	31.	187.97	{125., 243.}	506.355	0.771322	newly 5 weeks cumul	500.	1603.29	{1280., 1915.}	220.658	0.501188	newly 54 weeks cumul	536.	2226.12	{1618., 2749.}	315.321	0.609305
11	newly 3 weeks spec	22.	379.99	{323., 430.}	1627.23	1.23472	newly 3 weeks cumul	176.	1382.4	{1237., 1512.}	685.455	0.893623	newly 5 weeks spec	7.	242.4	{192., 288.}	3362.86	1.53423	newly 5 weeks cumul	192.	1927.99	{1693., 2149.}	904.161	0.999871	newly 53 weeks cumul	192.	2787.62	{2351., 3169.}	1351.89	1.15863
12	newly 3 weeks spec	117.	398.7	{351., 439.}	240.769	0.530253	newly 3 weeks cumul	568.	1427.01	{1318., 1579.}	151.234	0.398488	newly 5 weeks spec	54.	260.91	{222., 301.}	383.167	0.680127	newly 5 weeks cumul	704.	2011.08	{1831., 2187.}	185.665	0.454081	newly 53 weeks cumul	804.	3030.17	{2753., 3343.}	276.887	0.573677
13	newly 3 weeks spec	134.	342.52	{311., 376.}	155.612	0.406353	newly 3 weeks cumul	648.	1219.87	{1137., 1299.}	88.2515	0.274169	newly 5 weeks spec	83.	231.7	{203., 258.}	179.157	0.444082	newly 5 weeks cumul	810.	1734.1	{1602., 1856.}	114.086	0.329951	newly 50 weeks cumul	934.	2700.14	{2451., 2918.}	189.094	0.459955
14	newly 3 weeks spec	60.	294.11	{248., 349.}	390.183	0.686713	newly 3 weeks cumul	328.	1071.56	{953., 1240.}	226.695	0.511996	newly 5 weeks spec	20.	189.13	{155., 231.}	845.65	0.97078	newly 5 weeks cumul	376.	1497.23	{1326., 1756.}	298.199	0.597623	newly 52 weeks cumul	397.	2203.89	{1901., 2614.}	455.136	0.740753
15	newly 3 weeks spec	54.	290.77	{199., 378.}	438.463	0.716045	newly 3 weeks cumul	371.	1160.38	{856., 1446.}	212.771	0.485519	newly 5 weeks spec	11.	158.03	{93., 222.}	1336.64	1.13059	newly 5 weeks cumul	406.	1533.2	{1096., 1959.}	277.635	0.564898	newly 56 weeks cumul	423.	1969.74	{1283., 2712.}	365.66	0.649201
16	newly 3 weeks spec	69.	230.25	{185., 290.}	233.696	0.517606	newly 3 weeks cumul	368.	892.12	{755., 1047.}	142.424	0.380503	newly 5 weeks spec	29.	141.34	{108., 182.}	387.379	0.678288	newly 5 weeks cumul	440.	1218.18	{1033., 1462.}	176.859	0.437602	newly 52 weeks cumul	501.	1675.25	{1351., 2112.}	234.381	0.516861
17	newly 3 weeks spec	221.	379.27	{335., 417.}	71.6154	0.232682	newly 3 weeks cumul	873.	1304.43	{1150., 1410.}	49.4192	0.173112	newly 5 weeks spec	183.	272.81	{229., 309.}	49.0765	0.17026	newly 5 weeks cumul	1256.	1901.2	{1668., 2050.}	51.3694	0.17866	newly 52 weeks cumul	1842.	3147.31	{2586., 3434.}	70.8637	0.230113
18	newly 3 weeks spec	189.	356.93	{318., 400.}	88.8519	0.274313	newly 3 weeks cumul	833.	1245.76	{1143., 1347.}	49.551	0.17366	newly 5 weeks spec	133.	253.	{216., 294.}	90.2256	0.276597	newly 5 weeks cumul	1151.	1800.49	{1652., 1941.}	56.4283	0.193142	newly 50 weeks cumul	1580.	2936.1	{2625., 3382.}	85.8291	0.267019
19	newly 3 weeks spec	134.	279.15	{251., 304.}	108.321	0.317524	newly 3 weeks cumul	645.	958.66	{902., 1003.}	48.6295	0.171733	newly 5 weeks spec	78.	202.82	{176., 231.}	160.026	0.412692	newly 5 weeks cumul	830.	1400.13	{1299., 1474.}	68.6904	0.226626	newly 46 weeks cumul	1044.	2430.01	{2241., 2590.}	132.76	0.366213
20	newly 3 weeks spec	271.	264.73	{235., 295.}	7.30996	0.0325107	newly 3 weeks cumul	966.	861.71	{783., 941.}	10.8209	0.0507809	newly 5 weeks spec	160.	212.45	{189., 233.}	32.7813	0.121543	newly 5 weeks cumul	1369.	1313.71	{1201., 1417.}	6.03579	0.0272202	newly 46 weeks cumul	1954.	2713.42	{2474., 2901.}	38.8649	0.141669
21	newly 3 weeks spec	92.	326.91	{280., 384.}	255.337	0.547168	newly 3 weeks cumul	451.	1226.68	{1108., 1384.}	171.991	0.432854	newly 5 weeks spec	39.	195.59	{154., 239.}	401.513	0.694126	newly 5 weeks cumul	575.	1677.4	{1498., 1917.}	191.722	0.462929	newly 54 weeks cumul	717.	2332.41	{1958., 2783.}	225.301	0.508377
22	newly 3 weeks spec	126.	361.95	{236., 434.}	187.262	0.449862	newly 3 weeks cumul	547.	1222.31	{851., 1394.}	123.457	0.343282	newly 5 weeks spec	54.	263.75	{153., 331.}	388.426	0.674151	newly 5 weeks cumul	674.	1796.51	{1194., 2095.}	166.545	0.418341	newly 52 weeks cumul	743.	3029.55	{1767., 3725.}	307.746	0.596908
23	newly 3 weeks spec	180.	304.79	{250., 364.}	69.4722	0.222159	newly 3 weeks cumul	675.	1056.4	{857., 1233.}	56.9511	0.190878	newly 5 weeks spec	110.	217.	{160., 276.}	97.4	0.284962	newly 5 weeks cumul	921.	1532.85	{1245., 1771.}	66.633	0.215804	newly 51 weeks cumul	1186.	2507.24	{1764., 3122.}	111.403	0.31427
24	newly 3 weeks spec	117.	388.59	{346., 440.}	232.128	0.519481	newly 3 weeks cumul	561.	1416.31	{1307., 1584.}	152.462	0.401135	newly 5 weeks spec	36.	251.6	{220., 297.}	598.889	0.841223	newly 5 weeks cumul	658.	1977.98	{1825., 2226.}	200.605	0.476764	newly 52 weeks cumul	737.	2891.24	{2641., 3342.}	292.299	0.591766
25	newly 3 weeks spec	33.	406.01	{370., 441.}	1130.33	1.089	newly 3 weeks cumul	182.	1549.57	{1467., 1681.}	751.412	0.929423	newly 5 weeks spec	2.	242.08	{214., 272.}	12004.	2.08143	newly 5 weeks cumul	198.	2108.2	{2001., 2287.}	964.747	1.02658	newly 55 weeks cumul	198.	2907.35	{2744., 3118.}	1368.36	1.16622
26	newly 3 weeks spec	84.	380.71	{335., 443.}	353.226	0.653876	newly 3 weeks cumul	510.	1335.31	{1218., 1493.}	161.825	0.416879	newly 5 weeks spec	55.	260.52	{201., 309.}	373.673	0.670278	newly 5 weeks cumul	628.	1908.53	{1695., 2166.}	203.906	0.481239	newly 54 weeks cumul	701.	2920.33	{2387., 3478.}	316.595	0.615724
27	newly 3 weeks spec	183.	417.16	{353., 475.}	127.956	0.354677	newly 3 weeks cumul	804.	1530.38	{1301., 1673.}	90.3458	0.277556	newly 5 weeks spec	88.	264.23	{201., 312.}	200.261	0.470937	newly 5 weeks cumul	1035.	2129.25	{1772., 2351.}	105.725	0.310726	newly 55 weeks cumul	1231.	3038.43	{2432., 3469.}	146.826	0.387514
28	newly 3 weeks spec	70.	414.45	{363., 469.}	492.071	0.770206	newly 3 weeks cumul	412.	1516.7	{1378., 1677.}	268.131	0.564747	newly 5 weeks spec	15.	263.73	{218., 314.}	1658.2	1.24054	newly 5 weeks cumul	458.	2113.42	{1875., 2348.}	361.445	0.662548	newly 54 weeks cumul	486.	3040.95	{2595., 3509.}	525.71	0.793315
29	newly 3 weeks spec	27.	335.45	{307., 366.}	1142.41	1.09297	newly 3 weeks cumul	254.	1321.79	{1250., 1397.}	420.39	0.715766	newly 5 weeks spec	11.	192.43	{171., 214.}	1649.36	1.24015	newly 5 weeks cumul	278.	1769.67	{1682., 1868.}	536.572	0.803158	newly 56 weeks cumul	279.	2349.92	{2214., 2530.}	742.265	0.924311
30	newly 3 weeks spec	46.	381.99	{340., 430.}	730.413	0.917415	newly 3 weeks cumul	317.	1514.64	{1369., 1678.}	377.804	0.677982	newly 5 weeks spec	3.	221.23	{190., 253.}	7274.33	1.8652	newly 5 weeks cumul	342.	2026.82	{1840., 2243.}	492.637	0.771516	newly 56 weeks cumul	343.	2688.27	{2365., 3007.}	683.752	0.89261
31	newly 3 weeks spec	146.	312.88	{240., 377.}	114.301	0.325376	newly 3 weeks cumul	569.	1108.96	{912., 1267.}	94.8963	0.286244	newly 5 weeks spec	41.	208.9	{145., 264.}	409.512	0.696041	newly 5 weeks cumul	686.	1575.43	{1257., 1839.}	129.655	0.356382	newly 53 weeks cumul	786.	2367.29	{1736., 2983.}	201.182	0.469666
32	newly 3 weeks spec	142.	236.28	{191., 271.}	66.3944	0.217614	newly 3 weeks cumul	533.	785.43	{644., 870.}	47.3602	0.166051	newly 5 weeks spec	69.	184.95	{145., 218.}	168.043	0.422471	newly 5 weeks cumul	702.	1178.94	{966., 1289.}	67.9402	0.222563	newly 46 weeks cumul	971.	2339.52	{1785., 2694.}	140.939	0.37672
33	newly 3 weeks spec	184.	273.75	{238., 302.}	48.7772	0.170756	newly 3 weeks cumul	700.	915.86	{834., 976.}	30.8371	0.115933	newly 5 weeks spec	80.	210.32	{177., 241.}	162.9	0.416775	newly 5 weeks cumul	923.	1368.65	{1241., 1468.}	48.2828	0.170191	newly 46 weeks cumul	1232.	2561.67	{2191., 2858.}	107.928	0.315806
34	newly 3 weeks spec	186.	261.09	{226., 299.}	40.371	0.144365	newly 3 weeks cumul	603.	881.94	{787., 972.}	46.2587	0.163241	newly 5 weeks spec	102.	197.32	{158., 231.}	93.451	0.281621	newly 5 weeks cumul	831.	1305.	{1119., 1440.}	57.0397	0.193857	newly 46 weeks cumul	1130.	2385.72	{1941., 2760.}	111.126	0.320443
35	newly 3 weeks spec	127.	359.48	{272., 419.}	183.055	0.447137	newly 3 weeks cumul	520.	1290.02	{1004., 1459.}	148.081	0.391186	newly 5 weeks spec	58.	239.11	{168., 284.}	312.259	0.607997	newly 5 weeks cumul	660.	1824.57	{1372., 2086.}	176.45	0.437635	newly 53 weeks cumul	785.	2750.12	{1940., 3201.}	250.334	0.538565
36	newly 3 weeks spec	227.	346.92	{288., 393.}	52.8282	0.181289	newly 3 weeks cumul	850.	1184.27	{1062., 1317.}	39.3259	0.142209	newly 5 weeks spec	131.	256.1	{213., 294.}	95.4962	0.287168	newly 5 weeks cumul	1137.	1740.32	{1552., 1948.}	53.0624	0.182746	newly 49 weeks cumul	1502.	3004.2	{2531., 3361.}	100.013	0.297949
37	newly 3 weeks spec	162.	326.53	{235., 416.}	101.562	0.294555	newly 3 weeks cumul	656.	1130.01	{813., 1356.}	72.2576	0.229548	newly 5 weeks spec	79.	234.77	{144., 313.}	197.177	0.457898	newly 5 weeks cumul	838.	1643.89	{1161., 2011.}	96.1683	0.284798	newly 52 weeks cumul	1008.	2688.14	{1638., 3570.}	166.681	0.412382
38	newly 3 weeks spec	199.	383.54	{331., 433.}	92.7337	0.282849	newly 3 weeks cumul	840.	1357.33	{1238., 1479.}	61.5869	0.207241	newly 5 weeks spec	85.	261.91	{217., 302.}	208.129	0.485207	newly 5 weeks cumul	1088.	1938.93	{1771., 2125.}	78.2105	0.249598	newly 52 weeks cumul	1378.	3034.17	{2703., 3348.}	120.187	0.340713
39	newly 3 weeks spec	207.	306.69	{271., 345.}	48.372	0.169443	newly 3 weeks cumul	748.	1034.75	{931., 1144.}	38.4372	0.14001	newly 5 weeks spec	130.	227.24	{193., 257.}	74.8	0.239493	newly 5 weeks cumul	1035.	1530.55	{1365., 1689.}	47.8792	0.168429	newly 48 weeks cumul	1546.	2739.33	{2352., 3091.}	77.1882	0.246069
40	newly 3 weeks spec	211.	332.28	{293., 363.}	57.4787	0.195451	newly 3 weeks cumul	819.	1133.6	{1009., 1210.}	38.442	0.140094	newly 5 weeks spec	116.	241.52	{204., 267.}	108.207	0.316027	newly 5 weeks cumul	1057.	1658.01	{1467., 1771.}	56.86	0.194272	newly 48 weeks cumul	1430.	2851.98	{2526., 3128.}	99.4392	0.297976
41	newly 3 weeks spec	77.	396.42	{337., 465.}	414.831	0.708625	newly 3 weeks cumul	495.	1519.17	{1365., 1746.}	206.903	0.485158	newly 5 weeks spec	16.	237.93	{196., 295.}	1387.06	1.16709	newly 5 weeks cumul	559.	2064.52	{1825., 2376.}	269.324	0.56529	newly 56 weeks cumul	602.	2817.55	{2424., 3410.}	368.032	0.667099
42	newly 3 weeks spec	107.	318.44	{287., 345.}	197.607	0.472367	newly 3 weeks cumul	500.	1076.53	{1016., 1153.}	115.306	0.332459	newly 5 weeks spec	44.	233.92	{204., 270.}	431.636	0.722951	newly 5 weeks cumul	615.	1585.02	{1480., 1721.}	157.727	0.410441	newly 47 weeks cumul	696.	2757.28	{2496., 3075.}	296.161	0.596316
43	newly 3 weeks spec	71.	360.65	{319., 407.}	407.958	0.703877	newly 3 weeks cumul	380.	1352.03	{1238., 1522.}	255.797	0.549955	newly 5 weeks spec	40.	220.35	{186., 253.}	450.875	0.738059	newly 5 weeks cumul	453.	1856.98	{1701., 2078.}	309.929	0.611418	newly 55 weeks cumul	496.	2576.59	{2320., 2820.}	419.474	0.713799
44	newly 3 weeks spec	171.	309.41	{252., 360.}	80.9415	0.253348	newly 3 weeks cumul	617.	1048.51	{873., 1199.}	69.9368	0.227199	newly 5 weeks spec	118.	225.18	{168., 274.}	90.8305	0.274058	newly 5 weeks cumul	869.	1541.	{1249., 1768.}	77.3303	0.245189	newly 49 weeks cumul	1172.	2669.61	{2036., 3238.}	127.782	0.351362
45	newly 3 weeks spec	123.	328.03	{289., 363.}	166.691	0.424194	newly 3 weeks cumul	569.	1211.8	{1088., 1318.}	112.97	0.327011	newly 5 weeks spec	45.	206.07	{180., 234.}	357.933	0.658516	newly 5 weeks cumul	691.	1681.15	{1517., 1826.}	143.292	0.384884	newly 52 weeks cumul	789.	2441.18	{2201., 2621.}	209.402	0.489123
46	newly 3 weeks spec	168.	324.51	{219., 398.}	93.625	0.278808	newly 3 weeks cumul	709.	1083.06	{780., 1291.}	53.6784	0.181548	newly 5 weeks spec	88.	239.53	{173., 299.}	172.42	0.422462	newly 5 weeks cumul	909.	1601.67	{1100., 1921.}	76.8812	0.241165	newly 51 weeks cumul	1016.	2821.3	{1712., 3531.}	177.687	0.429344
47	newly 3 weeks spec	173.	335.74	{246., 424.}	94.0694	0.27859	newly 3 weeks cumul	651.	1134.79	{901., 1413.}	74.3149	0.234901	newly 5 weeks spec	105.	244.83	{177., 327.}	133.171	0.355995	newly 5 weeks cumul	904.	1666.47	{1305., 2104.}	84.344	0.258297	newly 52 weeks cumul	1045.	2809.3	{1928., 3726.}	168.833	0.417088
48	newly 3 weeks spec	241.	364.14	{318., 417.}	51.0954	0.177054	newly 3 weeks cumul	934.	1253.79	{1146., 1389.}	34.2388	0.126705	newly 5 weeks spec	108.	261.31	{220., 305.}	141.954	0.380548	newly 5 weeks cumul	1207.	1826.71	{1661., 2035.}	51.343	0.1786	newly 49 weeks cumul	1531.	3037.85	{2650., 3491.}	98.4226	0.295135
49	newly 3 weeks spec	134.	332.44	{252., 399.}	148.09	0.388316	newly 3 weeks cumul	470.	1138.11	{939., 1302.}	142.151	0.379958	newly 5 weeks spec	57.	236.44	{181., 291.}	314.807	0.607144	newly 5 weeks cumul	619.	1657.97	{1335., 1914.}	167.847	0.422616	newly 51 weeks cumul	792.	2774.95	{2012., 3388.}	250.372	0.534626
50	newly 3 weeks spec	82.	227.62	{187., 266.}	177.585	0.437824	newly 3 weeks cumul	351.	926.72	{800., 1039.}	164.023	0.418404	newly 5 weeks spec	30.	121.76	{86., 148.}	305.867	0.59775	newly 5 weeks cumul	410.	1216.52	{1033., 1368.}	196.712	0.468504	newly 56 weeks cumul	413.	1513.88	{1273., 1740.}	266.557	0.558555
